# The Effect of Varying Durations of Post-Harvest Cryogenic Treatments on the Quality of Cabernet Sauvignon Wines

**DOI:** 10.3390/foods14111972

**Published:** 2025-06-02

**Authors:** Zhihao Deng, Guo Cheng, Wangze Li, Pengfei Yang, Kekun Zhang, Keqin Chen, Yulin Fang

**Affiliations:** College of Enology, Heyang Viti-Viniculture Station, Ningxia Eastern Foot of Helan Mountain Wine Station, Northwest A&F University, Yangling 712100, China; 2019_dzh@nwafu.edu.cn (Z.D.); chengguoguozaizheli@nwafu.edu.cn (G.C.); 1073405864@nwafu.edu.cn (W.L.); yangpf2024@163.com (P.Y.); zhangkekun1990@nwafu.edu.cn (K.Z.)

**Keywords:** post-harvest grapes, low-temperature storage, wine quality, characteristic wine compounds, sensory quality

## Abstract

While cold chain transportation facilitates the utilization of wine grapes grown in remote mountainous areas, there is currently a lack of research on the impacts of different post-harvest temperatures on the quality of wine grapes. Therefore, three temperatures—room temperature (20 °C), chilled (4 °C), and frozen (−20 °C)—were selected to study the effects of post-harvest low-temperature treatments. The results indicated that the contents of tartaric acid and total polyphenols in the resulting wines were higher after the grapes underwent freezing, while the opposite trend was observed for those stored at room temperature. The changes in color lightness of wines were inversely correlated with the changes in color saturation and red chromaticity, while the yellow chromaticity of wines fermented after storage exhibited a slight increase. Rutin and ferulic acid were identified as the characteristic monophenols that decreased post-storage, and heptanal emerged as the volatile compound that decreased similarly. Furthermore, the tannin contents of the resulting wines demonstrated a strong correlation with temperature: when grapes were chilled, they reached the highest level, presenting a decreasing trend over time. For low-temperature storage, 1-hexanol, ethyl caprylate, isopentyl acetate, and (Z)-2-heptenal were identified as characteristic volatile compounds under the different treatments. Overall, the choice of an appropriate chilling temperature for the post-harvest storage of grapes can ensure the quality characteristics of the produced wine. This study confirms the potential value of cold chain transportation for the effective utilization of wine grapes grown in remote areas.

## 1. Introduction

In modern society, alcoholic beverages often play a significant role in social occasions. In the fruit wine sector, grapes have traditionally been the primary choice for wine production due to their high sugar content and rich secondary metabolites [1,2,3]. The quality and flavor of the produced wine are closely linked to the characteristics of the used grapes, making the cultivation and selection of grape berries integral to the winemaking process. Growing grapes necessitates careful management throughout every stage, including factors such as light, water, temperature, nutrients, and protection [4]. Consequently, substantial research has focused on optimizing relevant methods and understanding underlying mechanisms [5,6]. However, most studies have concentrated on the growth and development of grapes before ripening, while fewer investigations have examined changes in the fruit during ripening and after harvest. While research on other types of fruit has indicated that the quality, condition, and hormonal levels of fruit continue to evolve post-harvest and can be influenced by various factors [7,8,9], limited studies have addressed how the quality of grapes changes after harvest and the subsequent impacts on wine quality. For the wine industry, it is essential to further investigate the storage of fruit and the application of low temperatures, particularly in light of the disparities in production capacity among wineries in different regions and the demand for grape varieties and styles characteristic of certain areas.

Low temperatures typically inhibit the growth and development of plants; however, they can positively impact the preservation of fruit quality due to the effects of cold storage on post-ripening processes [10]. The effects of low temperatures on plant growth and development have been extensively studied in terms of molecular genetics, biochemistry, cultivation, and breeding [11,12,13,14], primarily focusing on enhancing plant resistance and regulating quality in response to increasingly harsh climatic conditions [15,16,17]. Additionally, the application of low temperatures in fruit storage is increasingly recognized for its ability to delay fruit aging and maintain quality [18], albeit with potential adverse effects on flavor compounds [19]; for instance, the flavonoid and anthocyanin levels—as well as the expression of associated genes—were significantly up-regulated in blood orange when stored at low temperatures (9 °C) [20,21]. Moreover, pineapple exhibited increased citric acid content and citrate synthase activity following low-temperature storage, leading to an increase in acidity [22]. The vitamin C content in mangoes diminishes with prolonged storage and ripening; however, lower storage temperatures can decelerate the ripening process and extend shelf life [23]. It is also crucial to consider the ripening status of fruit prior to cryogenic storage, as ripening continues throughout storage [24]. Furthermore, while cold storage has been shown to significantly enhance the visual appearance and soluble sugar content in strawberries, it adversely affects their anthocyanin levels [25]. Different fruits require distinct storage temperatures: the quality of Acerola cherries is best maintained at 10–12 °C, ensuring a firm texture and higher vitamin C content, while lower temperatures do not support optimal storage [26]. Conversely, apples stored near freezing temperatures can sustain their post-harvest quality, minimizing the degradation of organic acids [27]. Considering these and other results, storage temperature has become an important environmental factor for regulating the post-harvest quality of fruits.

Terroir characteristics are important factors affecting the quality of wine. In recent years, wine grapes have been gradually developed in remote mountainous areas due to their strong vitality and high economic value, exhibiting new product characteristics and enriching the sensory types of wine products. However, the complex geographical environments of these areas limit the transportation and direct brewing of wine grapes, which is not conducive to their long-term development. The development of cold chain transportation has provided new impetus for the production of wine grapes in special production areas, creating conditions for the efficient transportation of wine grapes. To date, there has been significant research on cold chain transportation regarding fruits such as peaches and blueberries [28,29]. Nevertheless, for wine grapes, there is currently limited research on the impacts of cold chain transportation temperatures on the quality of the resulting wine, with only research on the methods and varieties relevant to storage at low temperatures having been carried out [30], for example, regarding the application of sulfur dioxide or chitosan [31,32]. On the other hand, it remains unclear which temperatures are more suitable for the transportation of wine grapes (e.g., chilling or freezing temperatures). Based on the above, this study selected three temperatures—room temperature (20 °C), chilled (4 °C), and frozen (−20 °C)—in order to explore the effects of temperature on the quality changes in wine grapes during their post-harvest storage, providing a reference for the optimal selection of post-harvest cold chain transportation temperatures and storage times.

## 2. Materials and Methods

### 2.1. Grapes and Chemicals

This experiment utilized Cabernet Sauvignon grapes from the Helan Mountain East Foothill Appellation in Ningxia, vintage 2023. Analytical-grade reagents such as ethanol, sulfur dioxide, sodium chloride, ethyl acetate, and sodium acetate were sourced from Xilong Science Co. (Shantou, China). Chromatography-grade reagents, including methanol, acetic acid, and acetonitrile, were also obtained from Xilong Science Co. (Shantou, China). Additionally, 19 monomeric phenol standards, monomeric tannin standards, and 50 volatile compound standards were acquired from Aladdin Biochemical Technology Co. (Shanghai, China) and Macklin Biochemical Technology Co. (Shanghai, China). Deionized water was produced using an ultrapure water system at Northwest Agriculture and Forestry University (NWAFU, Yangling, China). HyperSep™ C18 solid-phase extraction columns (500 mg/6 mL) were purchased from Thermo Fisher Scientific (Waltham, MA, USA), and a 0.22 μm nylon filter membrane was sourced from Jinteng Experimental Equipment Co. (Tianjin, China).

### 2.2. Experimental Design and Vinification

After the grape materials were obtained, moldy or rotten berries were removed. The grapes were then randomly divided into three portions; taking one of them as an example, part of the day’s grapes were set aside for fermentation according to a small-scale procedure, which were labeled as ‘CK’. The remaining grape materials were stored in three groups for storage at different temperatures: 20 °C (room temperature), 4 °C (chilling), and −20 °C (freezing). The same division was performed for the other two portions of grapes. After 2 days (2D), 4 days (4D), and 6 days (6D) of storage, a portion of the grapes from each group were fermented using the aforementioned procedure, resulting in wine samples labeled as JJ (room temperature), LK (chilling), and BX (freezing), along with the corresponding time labels 2D, 4D, and 6D. All grapes were macerated with 30 mg/L pectinase (Lallzyme Ex, Lallemand, France) and clarified for 12 h. Fermentation was initiated using 200 mg/L of the same yeast (Lalvin RC212, Lallemand, France). Due to the variance in fermentation start times for each wine sample, maceration was halted by separating the skins when the specific gravity of the must reached 1.001. Fermentation was concluded by adding 50 mg/L SO_2_ when the specific gravity dropped below 0.994 and remained consistent during the subsequent fermentation. Finally, each portion of wine that had undergone the same condition as a biological replicate was stored at 4 °C until further evaluation.

### 2.3. Analysis of Physicochemical Parameters in Wine

In this experiment, the basic chemical parameters of the wine samples, including alcohol content, total acidity, tartaric acid, pH, soluble solids, and total phenols, were analyzed using Anton Paar’s FTIR Lyza 5000 Wine Automatic Analyzer (Anton Paar GmbH, Graz, Austria). The instrument was calibrated according to ISO 17025 [33] and operates based on the principles of Fourier Transform Infrared (FTIR) spectroscopy. In this method, the sample interacts with infrared light to generate an absorption spectrum. This spectrum is further processed through an interferometer system to create an interference pattern, which is then analyzed using a Fourier Transform to identify the corresponding chemical substances and determine their concentrations.

### 2.4. Analysis of Color in Wine

In this experiment, the color parameters of the wine samples were determined using the W100 Wine Color Analyzer (Vinventions SAS, Bordeaux, France). This instrument assesses the characteristics of wine colors according to the industry standard “Determination of the Color of Exported Wines CIE1976 (L*A*B) Color Space Method”. The analyzed parameters include L*, a*, b*, C*, h°, and ΔE, which are calculated automatically by the instrument’s software. Specifically, L* represents lightness, a* indicates reddish–greenish tones, b* denotes yellowish–blueish tones, C* signifies color saturation, h° symbolizes hue, and ΔE measures color difference [34].

### 2.5. Extraction and Analysis of Phenolic Compounds in Wine

In this experiment, the wine samples were extracted using ethyl acetate liquid–liquid extraction. Ten milliliters (10 mL) of wine samples were mixed with twenty milliliters (20 mL) of ethyl acetate and then centrifuged at 10,000 rpm for 5 min to facilitate extraction. The upper organic phase was collected, and this extraction process was repeated twice. The collected extracts were concentrated using a rotary evaporator at 30 °C and then dissolved in 10 mL of chromatographic methanol after drying. The samples were subsequently stored in a low-temperature environment and protected from light prior to analysis in the liquid phase. All samples were filtered through 0.22 μm microporous membranes.

The extraction of monomeric tannins from wine samples was performed using solid-phase extraction (SPE). Initially, the SPE column was activated with methanol, followed by cleaning with deionized water. Subsequently, 5 mL of wine samples were added to the column to adsorb the target compounds. The column was then washed three times with an equivalent volume of deionized water. Finally, elution was conducted with 5 mL of methanol twice, until the eluent from the column was colorless. The resulting eluate was evaporated to dryness at 30 °C under reduced pressure, dissolved in chromatographic methanol, and concentrated to a final volume of 5 mL to serve as the sample extract for liquid-phase analysis. All samples were filtered through 0.22 μm microporous filter membranes before analysis.

Monomeric phenols and monomeric tannins were analyzed using an Agilent 1260 Infinity II HPLC system (Agilent Technologies Inc., Santa Clara, CA, USA). Monomeric phenols were determined with a 2% acetic acid solution (mobile phase A) and acetonitrile (mobile phase B) as mobile phases, The solvent gradient elution was as follows: 0.00–5.00 min, 3–10% B; 5.00–15.00 min, 10–15% B; 15.00–33.00 min, 15–26% B; 33.00–40.00 min, 26–45% B; and 40.00–50.00 min, 45–3% B. The flow rate of the mobile phase was 1.0 mL/min, the column temperature was 30 °C, the detection wavelength was 280 nm, and the injection volume was 20 µL. Standard curves were constructed using 19 monomeric phenol standards through the external standard method, which are shown in Table S1. For the determination of monomeric tannins, a 1% acetic acid solution (mobile phase A) and methanol (mobile phase B) were employed as mobile phases, and the elution condition was 1.0 mL/min with 5% B for 10 min, a linear gradient from 5% to 20% B for 20 min, and a linear gradient from 20% to 40% B for 25 min. Then, before the next sample loading, the column was cleaned with 90% B for 10 min and equilibrated with 5% B for another 5 min, and the elution peak at 280 nm was monitored. A standard curve was generated via mass spectrometry following the tannin standards.

### 2.6. Extraction and Analysis of Volatile Compounds in Wine

The volatile compounds in wine were extracted using headspace solid-phase microextraction (HS-SPME). A mixture comprising 2 g of NaCl, 8 mL of the wine sample, and 10 μL of 4-methyl-2-pentanol (internal standard at 800 μg/L) was placed in a 20 mL sample vial. The vial was sealed and a solid-phase microextraction probe (ANPEL Laboratory Technologies Inc., Shanghai, China) was inserted. The extraction process was conducted at 42 °C for 30 min, followed by desorption in a gas chromatography injector at 250 °C for 5 min.

The specific determination method for volatile compounds referred to previously published methods [35]. The extracts were analyzed using a Thermo Fisher TRACE 1600 gas chromatograph coupled with an ISQ 7610 detector (Thermo Fisher Scientific Inc., San Jose, CA, USA) including a WAX polar capillary column (60 m × 0.25 mm, 0.25 μm). The temperature of the gas chromatography (GC) capillary column was programmed as follows: it was held at 50 °C for 2 min, increased to 220 °C at a rate of 3 °C/min, and held at this temperature for an additional 3 min. The carrier gas (helium) was maintained at a constant flow rate of 1 mL/min. The mass spectrometer operated in electron ionization (EI) mode with a mass range of 30–350 *m*/*z* and a resolution time of 8 min. The external standard quantification method was employed for the target compounds, and the quantitative analyses were monitored using 4-methyl-2-pentanol as an internal standard to ensure the stability and accuracy of the analytical process.

### 2.7. Sensory Evaluation of Wines

The Professional Sensory Training Team comprised 12 members, evenly split between 6 females and 6 males from Northwest Agricultural and Forestry University (NWAFU), aged 20 to 30 years. Each member possessed a relevant educational background and had undergone over two months of systematic training. To determine the sensory descriptors of aroma, we employed quantitative descriptive analysis as outlined in GB/T 16861-1997 (“Sensory Analysis: Identification and Selection of Descriptors for the Establishment of Sensory Profiles by Multivariate Analysis”) [36]. The sensory evaluation of wine samples was conducted online using the Simple Sensory Analysis System (v2.0) software developed by the China National Institute of Standardization (CNIS). Panelists recorded the sensory intensity of each aroma descriptor using a scale from 0 to 5, where 0 represented no sensation, 1 indicated weak, 2 slightly weak, 3 fair, 4 slightly strong, and 5 strong. They also assigned a subjective overall score (expressed in percentages) for each wine sample, as part of their preference judgment.

### 2.8. Statistical Analysis

One-way ANOVA, combined with Duncan’s multiple range test (*p* < 0.05), was used in SPSS 26 (IBM SPSS Inc., Chicago, IL, USA) to analyze differences among treatments. Additionally, *t*-tests were conducted using SPSS 26. The different letters in the figure indicate significant differences between the samples (*p* < 0.05), with the order of a–z (or A–Z) representing levels from high to low. Principal component analysis (PCA), hierarchical cluster analysis (HCA), two-tailed Pearson’s correlation test, and heat mapping were performed using Origin 9.1 (OriginLab Corporation, Northampton, MA, USA). Orthogonal partial least-squares discriminant analysis (OPLS-DA) was executed with SIMCA 14.1 (Umetrics, Umea, Sweden). All experiments were performed in triplicate, and results are presented as mean values ± standard deviation (SD).

## 3. Results and Discussion

### 3.1. Analysis of Basic Physicochemical Parameters of Wine Samples

**Figure 1:**
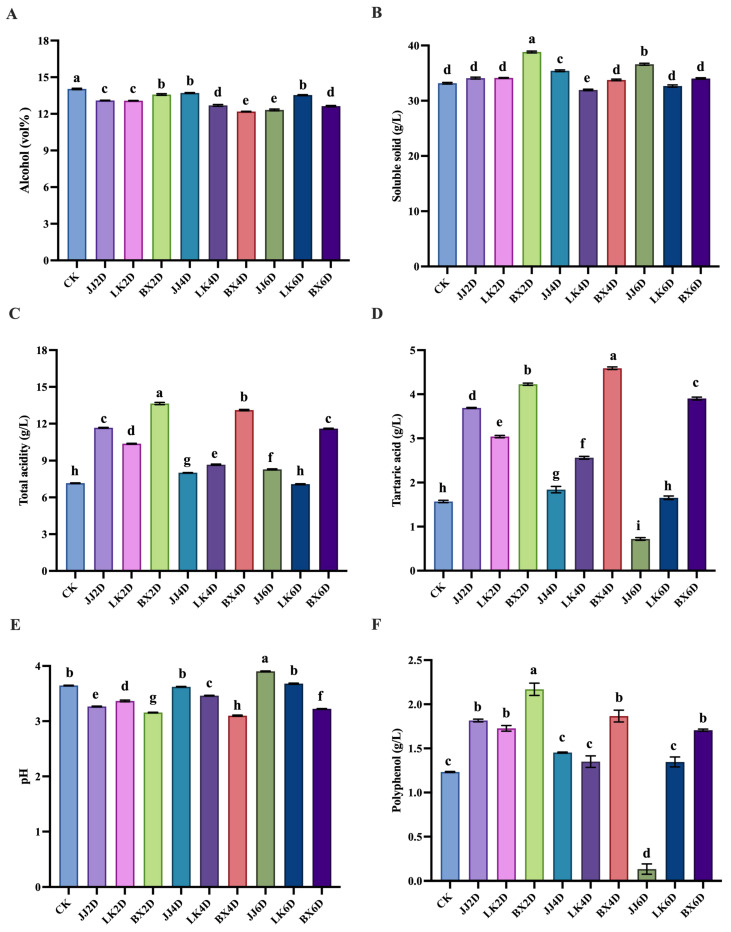
Physicochemical parameters in wine samples. (**A**) Contents of alcohol. (**B**) Concentrations of soluble solid. (**C**) Concentrations of total acidity. (**D**) Concentrations of tartaric acid. (**E**) Value of pH. (**F**) Concentrations of polyphenol. Different letters represent significant differences between wine samples according to Duncan’s test (*p* < 0.05).

Following the storage of grapes at various temperatures and durations, the basic physicochemical parameters of grapes were determined (as shown in Table S2). Fermentation was completed after an average of two weeks, and the basic physicochemical parameters are presented in Figure 1A–F. The alcohol content of all wine samples ranged from 12.19 to 14.04 vol% (Figure 1A), with no abnormalities observed during fermentation; the control (CK) group exhibited the highest alcohol content, which was significantly different from that of the other samples. The soluble solids content in all wine samples varied between 31.96 and 38.82 g/L, with the highest content found in the samples frozen for 2 days (BX2D) and the lowest in those chilled for 4 days (LK4D) (Figure 1B). Regarding total acidity, the BX group wine samples exhibited significantly higher levels than those of the other experimental groups, with titratable acidity (pH = 7.00) ranging from 11.59 to 13.64 g/L (Figure 1C). Notably, no experimental wine sample exhibited total acidity lower than that of the control group, with total acidity generally decreasing as storage time increased. Conversely, the content of tartaric acid—one of the most abundant and stable acids in wine [37]—in the BX samples (3.90–4.59 g/L) was significantly higher than that in the other experimental groups (Figure 1D). Furthermore, excluding the BX group, the tartaric acid contents in the JJ and LK groups showed a decreasing trend with longer grape storage times, suggesting potential degradation of tartaric acid during storage. As indicated in Figure 1E, the pH of the wine samples was also significantly influenced by tartaric acid content, exhibiting an inverse correlation; the sample stored at room temperature for 6 days (JJ6D) contained the lowest tartaric acid level (0.72 g/L) and the highest pH (3.90), when compared to the other samples. The increase in acidic substances might have been related to the balance of the glucose acid metabolism cycle in grapes, and the differences in the activities of enzymes related to sugar and acid metabolism at different temperatures might also have contributed to such results [38]. Polyphenols play a crucial role in determining the nutritional and functional characteristics of wine, affecting its color, aroma, flavor, and other sensory evaluation metrics. They comprise various organic molecules with complex structures and are vital indicators of wine quality. As depicted in Figure 1F, the total polyphenol content in the JJ6D wine samples (0.13 g/L) was significantly lower than that in all other experimental samples, indicating considerable loss of organoleptic and functional compounds under these storage conditions. In contrast, the total polyphenol contents in the remaining samples ranged from 1.23 to 2.17 g/L, with the BX group consistently presenting the highest overall values, warranting further investigation into their organoleptic properties. The observed increase in total polyphenols might have been due to the activation of secondary metabolic pathways in grapes induced by low-temperature storage, resulting in an increase in flavonoid-related substances [39]; however, under normal temperature conditions, these substances decreased with storage time, which might have been due to oxidation reactions [19].

### 3.2. Analysis of the Color of Wine Samples

**Figure 2:**
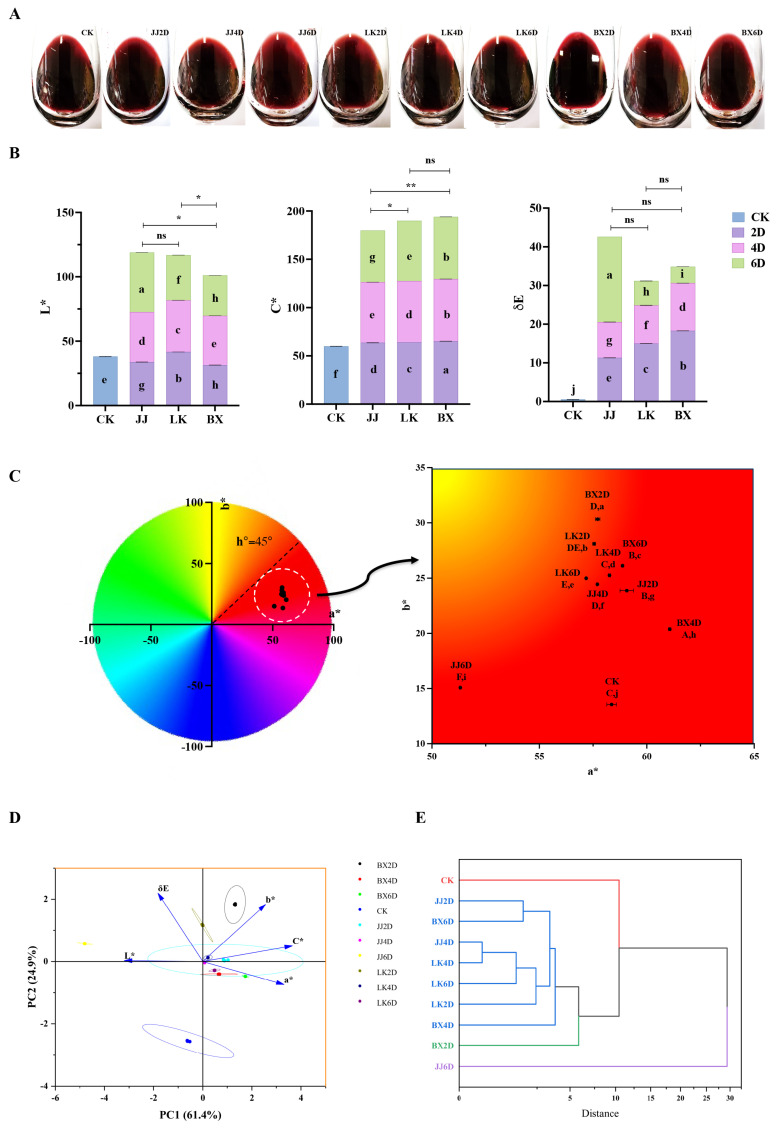
Color characteristics of wine samples. (**A**) Photographs of each wine sample in the glass. (**B**) The color index parameters of L*, C*, and ΔE values of the wine sample (the letters a–g indicated the significant differences in corresponding color index among all wine samples, *p* < 0.05). (**C**) The a*–b* value chromaticity distribution diagram. (**D**) PCA score diagram of all wine samples based on color characteristics. (**E**) HCA dendrogram based on color characteristics. * (*p* < 0.05) and ** (*p* < 0.01) represent significant differences between two treatments of the wine samples; ‘ns’ represents no significant difference.

The color of wine is the most immediate feature perceived by consumers once it has been poured into a glass, serving as a significant reflection of the wine’s attractiveness to them [40]. Therefore, assessing the effects of various treatments on the color of wine samples is crucial. The color-related index parameters for each wine sample, determined using the W100 Wine Color Analyzer, are illustrated in Figure 2A–E. Figure 2A displays a photograph of each wine sample in a wine glass, representing direct observations. Figure 2B shows the L*, C*, and ΔE values for each wine sample, and the L* value of CK was 38.09, placing it in the middle of the range for all samples (31.38–46.24). The sample exhibiting the highest L* value was JJ6D, while the lowest was from the samples frozen for six days (BX6D). Notably, the L* values of wine samples from the BX group were significantly lower than those from the JJ and LK groups. The C* value, which reflects the color saturation of each wine sample, represents the distance between the center of the chromaticity diagram and the sample point within the a*b* coordinate system. Generally, higher C* values indicate greater color saturation and vividness, enhancing consumer acceptance [41]. According to Figure 2B, JJ6D had the lowest C* value (at 53.49), which was significantly lower than that of CK (59.92). The samples with higher C* values were primarily from the BX group: specifically, BX2D (65.20), BX4D (64.38), and BX6D (64.42). In contrast, the overall C* values for the JJ group were significantly lower than those of the LK and BX groups. The ΔE value denotes the color difference between CK and other wine samples, and research indicates that human observers can distinguish color differences greater than three units [42]. As illustrated in Figure 2B, while ΔE values among the JJ, LK, and BX groups were not significantly different, the LK and BX samples displayed a trend of diminishing color difference from CK when the grapes were stored longer. Additionally, the JJ6D samples evidenced a distinct increase in color difference relative to CK, achieving the peak color difference among all samples. The a* and b* values indicate the two dimensions of color assessment in wine samples; namely, red–green and yellow–blue, respectively. Through employing the two-dimensional a*b* coordinate system, the hue of each wine sample could be accurately identified. All samples had a* values ranging from 51.32 to 61.07 and b* values ranging from 13.56 to 30.33. As shown in Figure 2C, the overall hue was characterized by a predominance of red with lighter yellow tones. Specifically, JJ6D exhibited the lowest a* value compared to the other samples and maintained the lowest b* value alongside CK, indicating a significant loss of red hue and an increase in yellow hue in JJ6D in comparison to all samples except itself.

To elucidate the primary effects of varying storage temperatures and durations on the color characteristics of wine samples, a principal component analysis (PCA) was conducted on each color indicator of the wine samples. As depicted in Figure 2D, the five color parameter indicators—L*, C*, ΔE, a*, and b*—were consolidated into two principal components, with PC1 and PC2 cumulatively explaining 86.35% of the variance in color changes among the wine samples. The parameters most significantly correlated with PC1 were L*, C*, and a*, indicating that brightness, saturation, and red hue comprised the main differences among the treated wine samples. Notably, C* and a* were positioned in the positive direction of PC1, while L* was in the negative direction, suggesting that brightness, saturation, and red hue exhibited contrasting trends. The parameters b* and ΔE were more strongly correlated with PC2 and were aligned in the positive direction of this component. The majority of other wine samples were positioned above CK indicating that, following grape storage treatment, the color differences and yellow hue of the wine samples exhibited similar changes. Additionally, in terms of PC1, the distribution of wine samples within the LK and BX groups was distinct and well-defined. Conversely, the hierarchical cluster analysis (HCA) based on the color indices, as represented in Figure 2E, revealed that samples JJ2D, JJ4D, LK2D, LK4D, LK6D, BX4D, and BX6D were clustered together, exhibiting significantly closer color performances when the Euclidean squared distance was less than 5. Similarly to the PCA findings, the overall color profile of JJ6D was notably different from that of the other wine samples.

### 3.3. Analysis of Phenolic Compounds in Wine Samples

**Figure 3:**
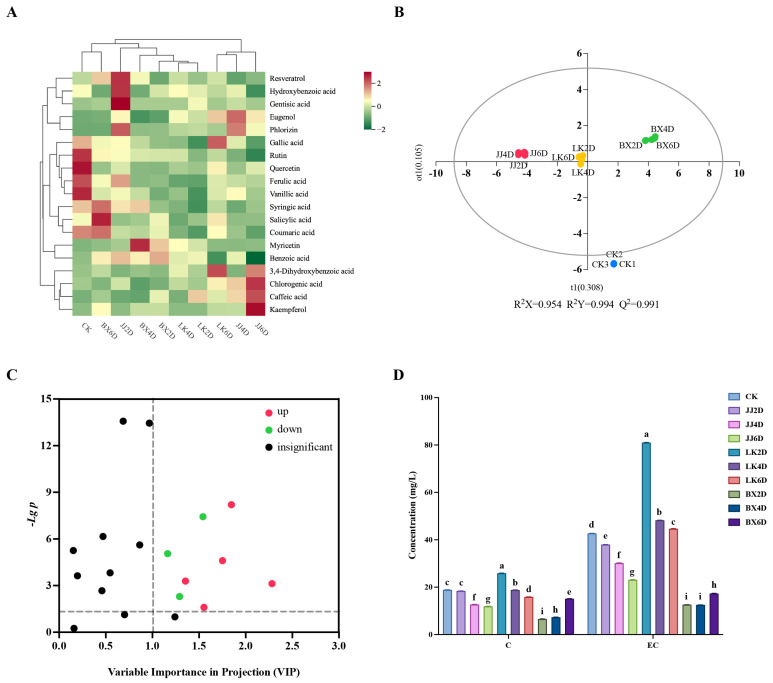
Differences in characterization of phenolic parameters in wine samples. (**A**) Clustered heat map of average concentrations of 19 monomeric phenolic compounds. (**B**) Results of OPLS-DA analysis based on monomer phenol content of wine samples. (**C**) Differential screening of monomeric phenolic compounds in CK compared to other wine samples (the dotted line indicated VIP = 1 and *p* = 0.05). (**D**) Concentrations of catechin (C) and epicatechin (EC) in wine samples. Different letters represented significant differences between same compound of wine samples according to Duncan’s test (*p* < 0.05).

Polyphenolic compounds are important components of wine, representing the secondary metabolites in grapes that significantly contribute to the organoleptic properties of wine, in addition to providing various health benefits. Using high-performance liquid chromatography (HPLC) for the analysis of wine samples, nineteen common monomeric phenolic compounds and two major tannin monomeric compounds were identified. As shown in Table S3, the contents of chlorogenic acid, salicylic acid, benzoic acid, and hydroxy formic acid were relatively high. Based on the measured phenolic content of each monomer in the wine samples, the cluster analysis results (Figure 3A) demonstrated that the strength of the correlation of monomer phenolic content between the wine samples was determined by the combination of storage temperature and time. All samples were divided into two main categories: one category included LK6D, JJ4D, and JJ6D, while the remaining samples formed the other. Notably, a stronger correlation existed between LK2D and LK4D; moreover, CK, JJ2D, and JJ6D had their own significant characteristics in terms of monomer phenolic content. The monomer phenolic contents of samples LK2D and LK4D were lower overall, with significantly lower levels of syringic acid, salicylic acid, and coumaric acid when compared to the other samples. Sample CK was characterized by higher levels of gallic acid, rutin, quercetin, ferulic acid, and vanillic acid, while sample JJ2D was characterized by higher levels of resveratrol, hydroxybenzoic acid, and gentisic acid. In contrast, JJ6D showed significantly higher levels of 3,4-dihydroxybenzoic acid, chloro-genic acid, caffeic acid, and kaempferol, while possessing lower levels of benzoic acid. The storage of grapes at varying temperatures resulted in distinct trends in the contents of monomeric phenols in the wines. In the JJ group, the levels of hydroxybenzoic acid, gentisic acid, phlorizin, gallic acid, rutin, ferulic acid, coumaric acid, and benzoic acid gradually decreased with increasing storage time. Conversely, the concentrations of chlorogenic acid, caffeic acid, and kaempferol gradually increased. In the LK group, LK6D notably differed from other wine samples, exhibiting significantly increased levels of eugenol, gallic acid, and 3,4-dihydroxybenzoic acid, as well as decreased levels of benzoic acid. The distribution of differences in the BX group with storage time were mainly reflected in the significantly higher levels of syringic acid, salicylic acid, and coumaric acid. Accordingly, the monophenols that increased with duration of storage were kaempferol and dihydroxybenzoic acid, while those that decreased included benzoic acid, phlorizin, and hydroxybenzoic acid. To further elucidate the effects of low-temperature storage on monomer phenols in wine and their associated characteristic compounds, a discriminative model (OPLS-DA) was simulated based on each monomer phenol compound, with the results displayed in Figure 3B. The JJ, LK, BX, and CK groups were distinctly separated, exhibiting a high degree of predictability (Q^2^ > 0.99) and strong fitting indices (R^2^X > 0.95, R^2^Y > 0.99). To identify the characteristic monomer phenols in each wine sample group, we screened all monomer phenolic compounds for differences based on VIP values (VIP > 1) and *t*-test *p*-values (*p* < 0.05). The results (Figure 3C) revealed eight key monomer phenolic compounds: chlorogenic acid, gentisic acid, benzoic acid, myricetin, eugenol, rutin, hydroxybenzoic acid, and ferulic acid. The trends for chlorogenic acid, gentisic acid, benzoic acid, myricetin, and eugenol were adjusted upwards in comparison to CK, while the trends for rutin, hydroxybenzoic acid, and ferulic acid were adjusted downwards. Within the experimental groups, only rutin and ferulic acid exhibited significant differences across all wine samples, suggesting that the more pronounced changes in monomeric phenols in wine samples derived from stored grapes were primarily characterized by decreases in rutin and ferulic acid. Furthermore, for the JJ group, there was a significant upward trend in chlorogenic acid, eugenol, and gentisic acid. In the LK group, only eugenol showed a significant increase, while the BX group displayed a significant decrease in hydroxybenzoic acid, along with significant increases in myricetin and benzoic acid. In summary, grapes stored in a chilled state exhibited relatively minor changes in wine monophenols, whereas storage in a frozen state led to significant alterations in the monophenols, particularly in terms of myricetin and benzoic acid.

Tannins are major contributors to the astringency of wine and play a critical role in stabilizing its color. Catechins and epicatechins are the primary monomers responsible for the variability of tannins [43]. Figure 3D illustrates the catechin and epicatechin levels measured in the wine samples. Notably, the total epicatechin content was greater than that of catechins, and a positive correlation was observed between the catechin and epicatechin levels in the samples, indicating that samples high in catechin also exhibited high levels of epicatechin. This correlation aided in the general characterization of the tannin content through catechin and epicatechin measurements. Among the three experimental groups, the BX group exhibited the lowest total tannin content, followed by the JJ group, while the LK group had the highest concentration. Neither of the contents in the BX nor the JJ groups exceeded the catechin content observed in the CK group, whereas samples from the LK group (specifically, LK2D and LK4D) demonstrated higher catechin levels than those in CK. Conversely, the epicatechin content was found to be greater than that in CK solely in the LK group samples, while the other two groups showed lower epicatechin content than CK. The analysis revealed a strong correlation between the tannin contents in the samples and the storage temperature of the grapes, with tannin content decreasing over prolonged storage duration. In comparison to CK, low-temperature storage conditions significantly enhanced the accumulation of tannins in wine, partially mitigating the degradation effects associated with extended storage.

### 3.4. Analysis of Volatile Compounds in Wine Samples

**Figure 4:**
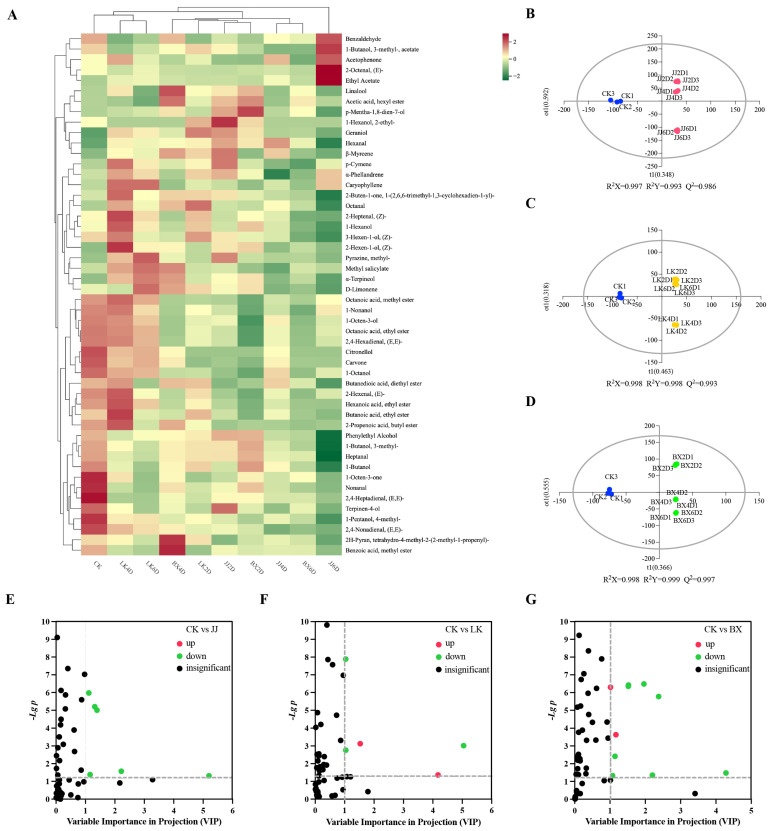
Differences in characterization of volatile compounds in wine samples. (**A**) Clustered heat map of average concentrations of 50 volatile compounds. (**B**–**D**) Results of OPLS-DA analysis of CK vs. JJ, CK vs. LK, and CK vs. BX based on volatile compounds content of wine samples. (**E**–**G**) Differential screening of volatile compounds in CK vs. JJ, CK vs. LK, and CK vs. BX (the dotted line indicated VIP = 1 and *p* = 0.05).

Volatile compounds are essential components in the aroma of a wine and serve as “fingerprints” that are associated with recognizable characteristics of a wine, such as its origin, variety, and age [44,45]. To further investigate the effects of low-temperature storage on the volatile compounds in wine samples and their trends, we measured a total of 50 volatile compounds using SPME-GC-MS, and the results are presented in Table S4. The analyzed volatile compounds included saturated fatty alcohols, aldehydes, aromatic alcohols, enols, esters, ketones, and other organic compounds. Due to the large number of volatile compounds and the need to compare the effects of different storage times and temperatures, hierarchical cluster analysis (HCA) facilitated the generation of Figure 4A, from which good parallelism in the results can be seen. Based on the levels and relevance of each volatile compound, we classified the wine samples into three categories: LK4D, LK6D, and CK as one group; JJ6D as an individual group; and the remaining wine samples as another group. Overall, the content and distribution of volatile compounds exhibited unique characteristics across the different sample groups, particularly for CK and JJ6D. The benzaldehyde, acetophenone, isoamyl acetate, trans-2-octenal, and ethyl acetate levels in JJ6D were significantly higher than in the other wine samples, whereas 1-butanol, phenethyl alcohol, heptanal, and 3-methyl-1-butanol levels in JJ6D were significantly lower. CK exhibited significantly higher ter-pinen-4-ol, 1-octen-3-one, nonanal, trans,trans-2,4-heptadienal, 4-methyl-1-pentanol, and trans,trans-2,4-nonadienal levels than other samples. Moreover, among the wine samples, CK contained a relatively high total sum of volatile compounds, while JJ6D had a relatively low total sum. When comparing the wine samples within each experimental group, the composition of various volatile compounds in the JJ group exhibited considerable variation with storage time, whereas octanol, 1-hexanol, damascenone, (Z)-2-heptenal, and 3-hexen-1-ol remained at relatively high levels in the LK group. Notably, higher levels of characteristic volatile compounds such as (Z)-2-hexen-1-ol, 2-methylpyrazine, methyl salicylate, and a-terpineol were present in LK4D and LK6D, while they were observed at lower levels in LK2D. The levels of volatile compounds such as 1-octen-3-ol, 1-nonanol, caprylic acid methyl ester, ethyl caprylate, and (E,E)-2,4-hexadienal in the BX group were significantly lower than those in the other experimental groups. Analyzing the trends of volatile compounds within each experimental group, the levels of 3-methyl-1-butanol, geraniol, and heptanal showed a gradual decrease over time, indicating a negative correlation with storage duration.

To further elucidate the differences in volatile compounds among the wine samples from each experimental group and identify the key compounds that contributed significantly to these differences, pairwise comparisons of the CK group with samples from other groups were conducted using Orthogonal Partial Least Squares-Discriminant Analysis (OPLS-DA). The results are presented in Figure 4B–D. All three sets of models demonstrated high predictability (Q^2^ > 0.9) and strong fit (R^2^X > 0.9, R^2^Y > 0.9). In the OPLS-DA model, CK was distinctly separated from wine samples in the JJ, LK, and BX groups. Differential volatile compounds and their characteristic compounds between each experimental group and CK were screened based on Variable Importance in Projection (VIP) values (VIP > 1) and *t*-test *p*-values (*p* < 0.05), with the results shown in Figure 4E–G. There were six differential volatile compounds identified between the CK and JJ groups—namely, heptanal, diethyl succinate, 1-octen-3-ol, trans-2-hexenal, 3-methyl-1-butanol, and ethyl hexanoate—all of which were down-regulated in the JJ group compared to CK. The CK and LK groups exhibited five differential volatile compounds: heptanal, 1-hexanol, ethyl acetate, 3-methyl-1-butanol, and 1-nonanal. In particular, 1-hexanol and ethyl acetate were up-regulated in the LK group relative to in CK, while the others were down-regulated. Additionally, there were ten differential volatile compounds between the CK and BX groups: heptanal, 1-octen-3-ol, diethyl succinate, trans-2-hexenal, ethyl caprylate, ethyl caproate, ethyl acetate, isoamyl acetate, (Z)-2-heptenal, and hexanal. In particular, ethyl acetate and hexanal exhibited up-regulation in the BX group in comparison to in CK, while the remaining compounds were down-regulated. In summary, heptanal was consistently lower in the experimental groups compared to in CK. Furthermore, the analysis of differential volatile compounds across the experimental groups indicated that the contents of certain volatile compounds varied uniquely with different storage temperatures. Specifically, a high level of 1-hexanol was characteristic of wines made from grapes stored in a chilled state, while low levels of ethyl caprylate, isoamyl acetate, and (Z)-2-heptenal were characteristic of wines produced from frozen grapes.

### 3.5. Analysis of Sensory Evaluation in Wine Samples

**Figure 5:**
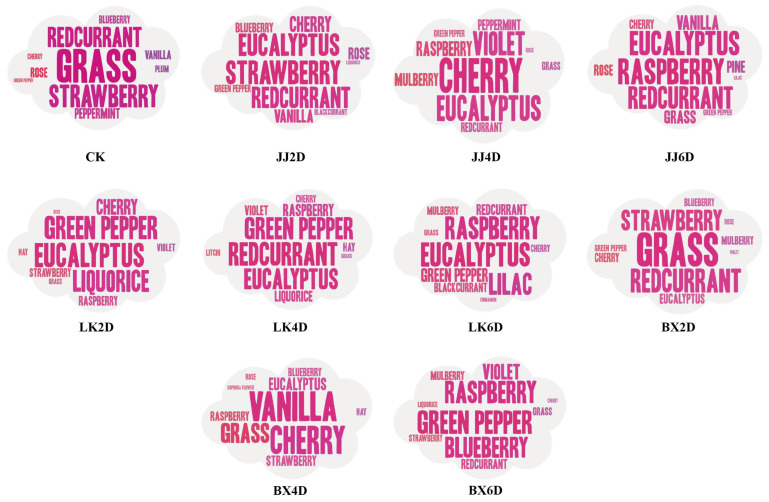
The top 10 characteristic aroma types ranked by sensory evaluation M-values for each wine sample. The font size from large to small indicates the M-value of each wine sample from high to low.

**Figure 6:**
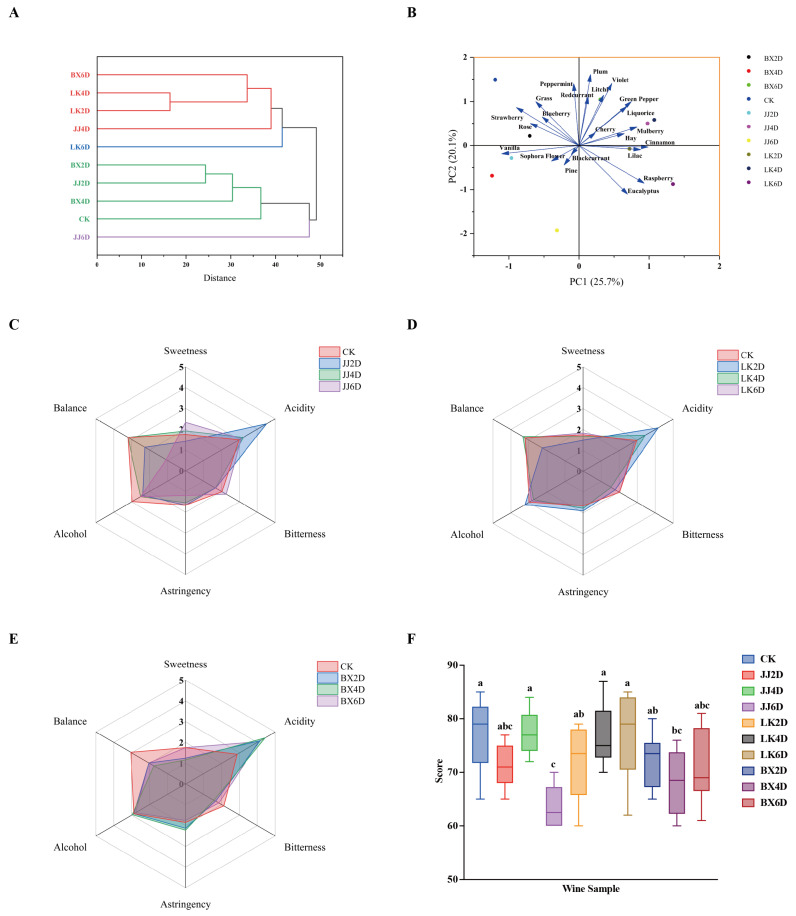
Analysis of sensory evaluation style of wine samples. (**A**) HCA dendrogram based on M-value of characteristic aroma types of wine samples. (**B**) PCA score diagram based on M-value of characteristic aroma types of wine samples. (**C**–**E**) Radar chart of six taste evaluations of CK vs. JJ, CK vs. LK, and CK vs. BX. (**F**) Overall sensory evaluation score of wine samples. Different letters represent significant differences between wine samples according to Duncan’s test (*p* < 0.05).

The organoleptic evaluation of each wine sample developed in this experiment was conducted by 12 professionally trained tasters. This evaluation included subjective assessments of aroma across 47 mainstream wine aroma types and 6 dimensions of taste. The tasters rated the intensity of the aromas and flavors on a scale from 0 to 5. Given the wide variety of aromas and the significant differences in characteristics among the experimental wines, we calculated the M-value of each aroma for each sample based on these scores. The top 10 aroma types with the highest M-value were identified, which were considered the main characteristic aromas of each corresponding wine sample for further analysis (Figure 5). Aggregating the characteristic aromas identified in the 10 wine samples, we selected a total of 22 mainstream aroma types that represented the characteristic aroma profiles for this study. The dominant aromas were botanical and red berry notes, complemented by floral and other varieties, with JJ6D being distinctive for its woody pine aroma compared to the other samples. Hierarchical cluster analysis (HCA) based on the M-values of different aromas across all samples yielded the results shown in Figure 6A, which indicate that when the squared Euclidean distance was less than 40, the experimental wine samples could be classified into four categories. The first category comprised LK2D, LK4D, BX6D, and JJ4D; the second included BX2D, JJ2D, BX4D, and CK; and LK6D and JJ6D each formed respective categories, with LK2D and LK4D exhibiting the highest similarity in cluster analysis. Notably, the classification results revealed that, after 6 days of storage, each wine sample group (including CK) developed distinct characteristic aromas; specifically, the JJ group presented woody aromas, the LK group showcased spicy notes, and the BX group was noted for green plant-like aromas. The results of principal component analysis (PCA), as presented in Figure 6B, allowed us to extract the characteristic aroma of each wine sample. The CK, BX2D, JJ2D, and BX4D categories were primarily characterized by grass, strawberry, and vanilla; LK2D, LK4D, JJ4D, and BX6D featured aromas such as green pepper, violet, and mulberry; LK6D was characterized predominantly by raspberry, eucalyptus, and cinnamon; and JJ6D was distinguished by pine and blackcurrant notes. While the characteristic aroma significantly contributes to defining the unique style of each wine sample, integrating taste sensations is essential for a more comprehensive sensory experience. For this purpose, a taste evaluation system was established, incorporating six dimensions: sweetness, acidity, bitterness, astringency, alcohol, and balance. The tasters used a five-point scale, and the mean values from their ratings for each sample were assigned as scores for the respective dimensions. When integrating the scoring data from the JJ, LK, and BX groups, we created the radar chart shown in Figure 6C–E. The chart illustrates that, in the JJ group, JJ4D’s taste profile was closer to that of CK, whereas JJ6D exhibited increased sweetness and bitterness, JJ2D had higher acidity, and CK revealed a more pronounced alcohol presence, consistent with the alcohol content of the samples. Within the LK group, LK6D’s taste profile closely approximated CK’s overall flavor; LK4D exhibited higher acidity and lower bitterness relative to both CK and LK6D; and LK2D was distinct from the others, showing the highest levels of acidity, alcohol, and astringency coupled with the lowest sweetness and balance scores. The BX group displayed considerable variation from CK, characterized by higher acidity, lower bitterness, and less balance, with BX6D presenting as sweeter and less astringent. Finally, to mitigate the influence of individual subjective preferences on the overall ratings—thereby reducing potential bias—we removed the highest and lowest scores from each wine sample’s overall evaluations, resulting in the outcomes depicted in Figure 6F. The analysis revealed that the highest-rated samples were CK, JJ4D, LK4D, and LK6D, with combined performance exceeding those of the other wine samples, while JJ6D received the lowest ratings. The results derived from the organoleptic evaluations provide clarity on the aromatic profiles, organoleptic characteristics, and overall assessments of each wine sample, facilitating a more rational understanding of the implications of post-harvest grape storage and the selection of optimal treatment methods.

### 3.6. Discussion

The basic physicochemical data of the samples indicated minimal variation in alcohol content, primarily attributed to the utilization of the same batch of grape berries. Furthermore, the storage of these grape berries at different temperatures and for varying durations did not significantly impact the fundamental sugar content. However, low-temperature treatments applied to different varieties of wine resulted in more pronounced differences [46]. Conversely, the acid levels—which are critical metabolites in wine—showed considerable variation with storage temperature; specifically, low temperatures—particularly those during freezing storage—led to increased acidity in the wine samples, as evidenced by elevated tartaric acid levels and decreased pH. This suggests that low temperatures may promote the formation and incorporation of acids (e.g., tartaric acid) into wine during both storage and fermentation processes [47], potentially benefiting wines that lack sufficient acidity [48]. Additionally, the total polyphenol content of the grapes was higher following frozen storage, likely as the frozen state offers better protection against the oxidative degradation of these polyphenols. In contrast, wine samples derived from grapes stored at room temperature exhibited increased oxidation over time. This was reflected in the gradual degradation of substances such as total polyphenols and tartaric acid, with a more significant decline compared to samples stored in refrigerated conditions. Regarding post-harvest grape polyphenols, Aloe Vera treatment has been shown to significantly increase the total phenolic content of fresh grapes stored after harvesting and correspondingly improved their antioxidant capacity [49]. Similarly, when treating grapes with salicylic acid, the total phenolic content in grape skins after harvesting also significantly increased and the storage time of grapes could be extended [50]. In this study, for grapes stored under low-temperature chilling, the physicochemical parameters remained close to those of the control (CK) wine samples, with reduced variability across indices as storage time increased, suggesting that low-temperature chilled storage was more effective in preserving the stability of the wines produced. In addition, soluble sugar and carbohydrate metabolism might play a crucial role in post-harvest regulation [51].

This study evaluated the influences of storage temperature and duration on the color quality of Cabernet Sauvignon wines. Brightness, saturation, and red chroma are critical indicators of color differences in wine samples, reflecting the effects of low-temperature storage on grape quality. The analysis of grapes stored under various conditions revealed distinct trends in color brightness; specifically, frozen storage resulted in reduced brightness compared to other methods, while the brightness of samples increased with extended storage at room temperature. These results suggest that storage temperature significantly affects the brightness of a wine’s color. Additionally, freezing grapes enhances the extraction of anthocyanins in the must [52], which can increase color saturation and enhance visual perception [53]. Conversely, room-temperature storage may negatively affect color saturation, diminishing the wine’s appeal. Therefore, the optimal choice of storage temperature and duration requires balancing brightness and saturation, with chilled storage appearing to offer a more favorable compromise. In the red color assessment, the a* values for all samples except for JJ6D were clustered together. Principal component analysis (PCA) indicated that samples other than JJ6D were positioned to the right of CK, suggesting abnormal color behavior in the JJ6D samples and emphasizing the need for its separate analysis. The chromaticity angle (h°) of 45° demarcated the region occupied by the red samples, and h° values for other samples increased in relation to CK, indicating a potential trend of color alteration in grapes following storage.

The results of the monomeric phenol determination in the experimental wine samples revealed stronger correlations among the experimental groups stored at low temperatures, particularly for samples LK2D and LK4D as well as BX2D and BX4D. This indicates that, when the grapes were stored at low temperatures, the formation of specific characteristics of the monomeric phenolics in the resulting wine samples was promoted. Furthermore, the low-temperature storage of the grapes for up to four days rendered the monomeric phenolics in the wine samples insensitive to the duration of storage. In contrast, the correlation observed between LK6D and BX6D suggests that the impact of low-temperature storage on the monomeric phenols in the wine samples diminished significantly after a storage period extending to six days. Additionally, gallic acid, rutin, quercetin, ferulic acid, and vanillic acid exhibited significantly higher concentrations in the CK group when compared to other wine samples. The OPLS-DA results further indicated that rutin and ferulic acid are characteristic monomeric phenolic compounds that decrease due to storage, suggesting that these compounds can serve as a reference to assess the storage status of raw grapes. Moreover, the characteristics of the monomeric phenolic compounds found in the wine samples of each experimental group could assist in determining the temperature conditions under which the grapes were stored. Regarding the evolution of tannin monomers across the experimental groups, the lower tannin levels in the BX group may be attributable to the fermentation process, which involved freezing the grapes and consequently hindered the incorporation of compounds such as catechins or epicatechins into the wine [54,55]. This resulted in lower tannin levels, independent of the storage duration. Therefore, when integrating the phenolic determinations for each wine sample, the LK group samples demonstrated relatively higher levels of tannins and less variation in other monomeric phenols, comprising more suitable conditions for the storage of grape berries. In addition to temperature-related conditions, exogenous adenosine triphosphate has also been shown to increase the contents of flavonoids and phenolic substances in post-harvest grapes [56].

The results of the volatile compound measurements in all wine samples indicated that the CK samples contained relatively high amounts of volatile compounds, whereas the JJ6D samples exhibited relatively low levels. This trend suggests that the overall level of volatile compounds in the wine samples decreased when grapes were stored for extended periods in their regular state. Additionally, the BX6D samples presented the lowest overall levels of volatile compounds within the BX group, which may be associated with flavor loss due to low-temperature storage [57]. In contrast, the volatile compounds in the LK group did not uniformly decrease with storage time compared to other experimental groups, particularly in the case of octanol, 1-hexanol, damascenone, (Z)-2-heptenal, and 3-hexen-1-ol. This finding suggests that chilling grapes at low temperatures at least partially mitigates the loss of volatile compounds in wines. Furthermore, the concentrations of 3-methyl-1-butanol, geraniol, and heptanal in the wine samples across all experimental groups demonstrated a significant decreasing trend with increasing storage time. This may reflect the differences in the characteristics of the wines resulting from storing grapes for differing durations, thus providing insight for optimization of the storage duration. Meanwhile, heptanal was significantly reduced in all experimental wine groups compared to in CK after OPLS-DA, indicating it to be a characteristic volatile compound which is decreased in wines after the storage of grapes. In conclusion, chilling grapes reduces the loss of volatile compounds in wine, helping to maintain stability and favoring the production of high-quality wines during grape storage.

The indicators measured in wine samples significantly influence both the physical properties and functionality of the wine and are crucial for guiding organoleptic evaluations [58]. The organoleptic tastings in this study revealed a preference among the professional tasters for the CK, JJ4D, LK4D, and LK6D samples, suggesting that low-temperature treatments can positively affect organoleptic evaluations, to some extent [59]. As basic physicochemical indicators (e.g., alcohol content) do not consistently impact aroma or flavor intensity [60], the characteristic aroma type of each wine sample serves as a more accurate representation. The grouping of the BX2D, BX4D, and JJ2D samples with CK demonstrated that the storage of grapes under freezing conditions resulted in wines with insignificant changes in their characteristic aroma types, when compared to those that were not stored. Conversely, the woody aroma of JJ6D was likely due to oxidation, as all samples were unaged, and final organoleptic tasting scores indicated that the defects in the JJ6D samples were more pronounced.

Overall, the low-temperature treatment of post-harvest grapes was found to be conducive to the accumulation of phenolic and volatile compounds in the resulting wine, as well as improved sensory quality. At the same time, the quality of the wines produced varied with different storage times, providing a technical reference for screening suitable parameters and improving the utilization efficiency of wine grapes in remote areas through cold chain transportation in order to produce high-quality wine. Although we identified the potential application value of cold chain transportation for the wine grape industry in mountainous areas, further exploration is needed for the selection of more refined cold chain transportation temperatures, as well as the associated humidity environment, packaging materials, and storage methods.

## 4. Conclusions

In summary, the storage of grapes at low temperatures—particularly in a frozen state—contribute to increased acidity in wine, promoting higher levels of tartaric acid and total polyphenols. While this results in a more saturated color, it comes at the expense of brightness. Additionally, the aromas tend to exhibit green plant notes, although the low tannin content of such wines represents a notable flaw. Storing grapes at room temperature for extended periods leads to a significant reduction in both tartaric acid and total polyphenol contents, accompanied by a loss of the characteristic red and yellow hues in the wine. A major drawback of this method is the pronounced loss of volatile compounds over time, which may result in the introduction of woody aromas. Conversely, the storage of grapes under chilled conditions resulted in physicochemical indices and color profiles similar to those of control samples (i.e., without storage), with only minor changes in the contents of individual monomer phenols. Furthermore, organoleptic evaluations indicated an increase in spicy aroma characteristics and favorable tannin accumulation and a reduced decline in volatile compounds with a prolonged storage time. Therefore, storing grapes at 4 °C under chilled conditions is more likely to preserve the overall quality of the produced wine, thus providing a technical reference for selecting suitable storage temperatures and periods in the context of cold chain transportation. Of course, in practical production applications, it is also necessary to consider the balance between the production revenue derived from the wine grapes and the cost of cold chain transportation.

## Data Availability

The original contributions presented in this study are included in the article/Supplementary Material, and further inquiries can be directed to the corresponding author.

## Supplementary Materials

The following supporting information can be downloaded at https://www.mdpi.com/article/10.3390/foods14111972/s1. Table S1: Calibration curve of quantified phenolic compounds in wine obtained by HPLC; Table S2: Physicochemical parameters in grape samples; Table S3: Compositions and concentrations of phenolic monomers in wine samples (mg/L); Table S4: Compositions and concentrations of volatile compounds in wine samples (μg/L).
