# Supplementary material for: The Effect of Varying Durations of Post-Harvest Cryogenic Treatments on the Quality of Cabernet Sauvignon Wines
Source: Foods. 2025 Jun 2;14(11):1972. doi: 10.3390/foods14111972 (PMC12154554; doi:10.3390/foods14111972)
Supplement: Supplementary file 1 [file foods-14-01972-s001.zip › foods-3569190-supplementary.pdf]

Supplementary data for

The Impact of Varying Durations of Post-Harvest Cryogenic Treatments on the Quality of Cabernet Sauvignon Wines

Zhihao Deng, Guo Cheng, Wangze Li, Pengfei Yang, Kekun Zhang, Keqin Chen*, Yulin Fang*

College of Enology, Shaanxi Provincial Key Laboratory of Viti-Viniculture, Viti-viniculture Engineering Technology Center of State Forestry and Grassland Administration, Shaanxi Engineering Research Center for Viti-Viniculture, Heyang Viti-viniculture Station, Ningxia Eastern Foot of Helan Mountain Wine Station, Northwest A&F University, Yangling, 712100, China

* Corresponding author.

E-mail address: fangyulin@nwafu.edu.cn (Y. Fang), chenkeqin1985@nwafu.edu.cn (K. Chen)

Supplementary Table Captions

Table S1. Calibration curve of quantified phenolic compounds in wine obtained by HPLC.

Table S2. Physicochemical parameters in grape samples.

Table S3. Compositions and concentrations of phenolic monomers in wine samples (mg/L).

Table S4. Compositions and concentrations of volatile compounds in wine samples (μg/L).

Table S1. Calibration curve of quantified phenolic compounds in wine obtained by HPLC.

Number	Standard name	Retention time (min)	Standard curve	R^2
1	Resveratrol	50.284	Y=0.0282X+0.7257	0.9997
2	Chlorogenic acid	26.947	Y=0.065X+4.7105	0.995
3	Rutin	42.651	Y=0.1412X+1.3262	0.9997
4	Caffeic acid	30.260	Y=0.0262X+1.3684	0.9988
5	Kaempferol	62.355	Y=0.0568X+1.0325	0.9998
6	Gallic acid	6.056	Y=0.0282X+1.3779	0.9891
7	Quercetin	54.265	Y=0.0657X+1.9461	0.9994
8	Coumaric acid	38.633	Y=0.0155X+1.2872	0.9993
9	Myricetin	19.312	Y=0.00028X-0.1397	0.9996
10	Vanillic acid	29.665	Y=0.0666X+1.5124	0.9994
11	Ferulic acid	41.725	Y=0.0408X+2.1659	0.9994
12	Syringic acid	33.520	Y=0.0329X+2.9879	0.9997
13	Hydroxybenzoic acid	12.827	Y=0.0635X+2.8767	0.9998
14	Salicylic acid	46.411	Y=0.1388X+5.5002	0.9991
15	Phlorizin	48.659	Y=0.0459X-0.888	0.9998
16	3,4-Dihydroxybenzoic acid	3.229	Y=0.0002X-0.0047	0.9999
17	Gentisic acid	5.445	Y=0.00026X+0.0571	0.9999
18	Eugenol	22.877	Y=0.00028X-0.0802	0.9997
19	Benzoic acid	14.269	Y=0.00081X+0.3382	0.9999
20	Catechin	26.364	Y=0.1616X+1.6246	0.9995
21	Epicatechin	27.674	Y=0.1464X+6.2757	0.9997

Note: X denotes peak area (mAU·S), and Y denotes substance concentration (mg/L).

Table S2. Physicochemical parameters in grape samples.

Grapes sample	CK	JJ2D	LK2D	BX2D	JJ4D	LK4D	BX4D	JJ6D	LK6D	BX6D
Total sugar (°Brix)	25.04±0.07h	24.45±0.1g	24.10±0.03f	24.51±0.35g	22.62±0.04c	23.25±0.03d	23.75±0.18e	18.79±0.11a	21.41±0.02b	22.39±0.07c
Titratable acidity (g/L)	3.47±0.10ab	3.48±0.04ab	3.40±0.02a	3.59±0.08b	3.78±0.05c	4.85±0.08d	6.38±0.19f	3.62±0.03b	5.75±0.09e	7.19±0.05g
Glucose (g/L)	120.86±2.74 g	116.68±0.75 ef	116.39±1.63 ef	117.76±0.90 f	108.26±1.51 b	112.54±0.82 cd	114.90±0.70 de	97.84±2.42a	106.68±0.10 b	112.04±1.08 c
Fructose (g/L)	130.94±2.11 f	126.60±1.65 e	127.95±0.77 e	126.50±1.65 e	111.75±2.11 b	120.26±0.60 cd	122.89±0.85 d	98.89±3.59a	114.56±0.47 b	117.95±1.18 c
Tartaric acid(g/L)	2.52±0.04cd	2.53±0.11cd	2.44±0.05c	2.60±0.08d	2.17±0.03b	2.81±0.07e	3.31±0.04g	1.95±0.05a	3.15±0.05f	4.42±0.08h
Malic acid(g/L)	2.20±0.05c	2.18±0.04c	2.19±0.02c	2.18±0.03c	1.88±0.08b	2.21±0.05c	2.98±0.02e	1.58±0.03a	2.29±0.03d	3.58±0.05f
pH	4.62±0.04g	4.39±0.04f	4.38±0.03f	4.37±0.01f	4.27±0.03e	4.11±0.02d	4.08±0.02d	3.95±0.02c	3.84±0.01b	3.62±0.01a

Note: Different letters represent significant differences between the grape samples according to Duncan's test ($p < 0.05$).

Table S3. Compositions and concentrations of phenolic monomers in wine samples (mg/L).

phenolic monomers	Wine samples									
	CK	JJ2D	JJ4D	JJ6D	LK2D	LK4D	LK6D	BX2D	BX4D	BX6D
Gallic acid	1.58±0.00h	1.52±0.00g	1.50±0.00e	1.40±0.00b	1.39±0.00a	1.44±0.00c	1.64±0.00i	1.45±0.00c	1.48±0.00d	1.51±0.01f
Hydroxybenzoic acid	8.86±0.02h	15.14±0.00j	8.20±0.01g	3.29±0.00a	8.14±0.02f	8.93±0.01i	7.05±0.01d	7.68±0.03e	4.76±0.01c	4.69±0.00b
Chlorogenic acid	15.49±0.04d	18.58±0.01f	25.34±0.02h	35.64±0.05i	15.48±0.00d	17.42±0.03e	22.83±0.01g	12.62±0.00b	10.61±0.01a	13.19±0.01c
Vanillic acid	2.15±0.01j	1.84±0.00h	1.86±0.00i	1.73±0.00d	1.60±0.00a	1.71±0.00c	1.80±0.00g	1.66±0.00b	1.77±0.00e	1.80±0.00f
Caffeic acid	3.64±0.00c	3.55±0.01b	5.17±0.00g	6.34±0.01i	5.27±0.01h	3.65±0.00c	4.51±0.00e	4.59±0.01f	3.47±0.00a	4.03±0.01d
Syringic acid	3.52±0.00i	3.45±0.00g	3.10±0.00b	3.20±0.00e	3.01±0.00a	3.14±0.00c	3.26±0.00f	3.18±0.00d	3.50±0.01h	3.70±0.00j
Coumaric acid	1.40±0.00f	1.36±0.00c	1.35±0.00b	1.34±0.00a	1.34±0.00a	1.35±0.00b	1.38±0.00e	1.37±0.00d	1.36±0.00c	1.41±0.00g
Ferulic acid	5.23±0.02j	4.55±0.01i	3.27±0.01f	3.12±0.01e	2.42±0.00a	2.46±0.00b	3.44±0.01g	2.80±0.01d	2.74±0.00c	3.66±0.01h
Rutin	9.84±0.00i	5.62±0.00g	3.08±0.04b	1.60±0.00a	3.28±0.03c	4.81±0.00e	4.45±0.02d	4.84±0.01e	4.96±0.01f	5.93±0.02h
Salicylic acid	8.78±0.01e	8.05±0.01b	8.01±0.01b	8.03±0.01b	8.08±0.01b	7.75±0.00a	9.05±0.01f	8.72±0.06d	8.16±0.01c	10.54±0.04g
Phlorizin	0.47±0.07a	2.62±0.01g	2.37±0.10f	1.38±0.13e	1.03±0.00d	0.98±0.01d	0.85±0.01c	0.72±0.01b	0.69±0.00b	0.47±0.01a
Resveratrol	0.81±0.00e	0.96±0.00i	0.76±0.00a	0.78±0.00c	0.79±0.00d	0.82±0.00f	0.82±0.00f	0.77±0.00b	0.84±0.00g	0.87±0.00h
Quercetin	4.33±0.02i	2.51±0.00f	2.13±0.00d	2.55±0.01g	2.13±0.00d	2.20±0.00e	2.87±0.00h	1.97±0.00a	2.00±0.00b	2.11±0.00c

phenolic monomers	Wine samples									
	CK	JJ2D	JJ4D	JJ6D	LK2D	LK4D	LK6D	BX2D	BX4D	BX6D
Kaempferol	0.00±0.00a	0.00±0.00a	0.11±0.01b	0.71±0.03d	0.00±0.00a	0.00±0.00a	0.10±0.01b	0.11±0.01b	0.11±0.00b	0.23±0.02c
Eugenol	1.74±0.06c	4.61±0.01f	7.31±0.12i	4.89±0.17g	3.43±0.07d	4.25±0.07e	5.50±0.05h	1.35±0.02b	0.94±0.01a	1.84±0.01c
3,4-Dihydroxybenzoic acid	0.38±0.02c	0.00±0.00a	0.00±0.00a	3.82±0.13g	0.71±0.04d	1.55±0.03f	4.62±0.11h	0.17±0.01b	0.36±0.10c	0.95±0.05e
Benzoic acid	6.98±0.10c	14.46±0.23f	10.63±0.41d	0.75±0.03a	10.89±0.09d	10.30±1.34d	3.69±0.13b	15.26±0.00f	12.30±0.11e	12.42±0.06e
Gentisic acid	0.00±0.00a	18.26±0.47e	2.12±0.12c	0.37±0.01b	4.51±0.20d	0.54±0.07b	0.41±0.03b	0.60±0.04b	0.57±0.04b	0.68±0.03b
Myricetin	0.40±0.00a	1.60±0.00d	0.72±0.06b	0.83±0.06c	3.06±0.03e	4.60±0.03f	0.74±0.09b	6.45±0.03g	11.63±0.03h	0.90±0.08c

Note: Different letters represent significant differences between the wine samples according to Duncan's test ($p < 0.05$).

Table S4. Compositions and concentrations of volatile compounds in wine samples (μg/L).

Volatile compounds	CAS	LRi	Wine samples									
			CK	JJ2D	JJ4D	JJ6D	LK2D	LK4D	LK6D	BX2D	BX4D	BX6D
2-Hexenal, (E)-	6728-26-3	1230	728.97±2.70i	338.74±7.23c	473.57±7.45f	437.06±14.75e	574.34±6.82h	823.86±2.41j	496.24±3.38g	237.23±7.53a	375.50±1.47d	293.91±3.87b
1-Hexanol	111-27-3	1372	6140.08±113.31f	5073.61±32.32d	5367.04±37.41e	1554.38±8.80a	7904.43±91.83h	10782.50±89.68i	6926.32±84.46g	7906.90±5.11h	4653.05±35.66c	3041.15±37.91b
Octanoic acid, methyl ester	111-11-5	1374	27.95±0.81d	25.14±0.12b	25.68±0.16b	26.37±0.27c	25.28±0.03b	28.67±0.26e	27.62±0.08d	24.17±0.02a	25.08±0.10b	25.07±0.09b
2-Hexen-1-ol, (Z)-	928-94-9	1407	0.00±0.00a	1.68±0.08cd	0.00±0.00a	0.00±0.00a	0.58±0.06b	3.87±0.57e	1.52±0.11c	1.92±0.11d	1.48±0.20c	0.63±0.01b
2,4-Hexadienal, (E, E)-	142-83-6	1414	73.57±3.62g	43.00±1.06d	48.63±0.43e	43.93±0.75d	47.90±0.54e	72.17±0.08g	63.94±1.42f	23.33±0.06a	36.76±0.32c	33.68±0.24b
Octanoic acid, ethyl ester	106-32-1	1440	344.00±26.42g	159.41±0.89c	193.00±7.86d	177.62±10.56d	191.03±1.46d	326.59±6.43f	285.80±3.60e	41.42±0.08a	116.74±1.79b	101.63±0.30b
Butanedioic acid, diethyl ester	123-25-1	1677	2091.50±50.33g	862.46±1.70b	1936.37±24.56e	546.59±11.45a	2008.29±1.88f	2286.66±41.24h	1035.98±6.10c	1057.89±43.26c	2039.29±43.00f	1384.38±2.05d
Acetophenone	98-86-2	1694	15.18±0.05e	13.74±0.04b	16.22±0.18f	17.52±0.17g	14.89±0.09d	16.45±0.13f	14.85±0.09d	13.71±0.30b	14.18±0.20c	13.32±0.04a

Volatile compounds	CAS	LRi	Wine samples									
			CK	JJ2D	JJ4D	JJ6D	LK2D	LK4D	LK6D	BX2D	BX4D	BX6D
Butanoic acid, ethyl ester	105-54-4	1041	165.18±2.92g	95.71±0.49d	121.12±2.46f	50.85±0.69b	179.20±5.82h	262.41±5.09i	108.80±2.49e	43.55±0.47a	97.40±0.91d	67.79±1.87c
Hexanal	66-25-1	1097	226.83±11.71b	411.36±3.17e	420.88±3.18e	183.47±2.20a	353.76±12.21d	353.17±2.47d	329.52±4.65c	361.80±0.86d	337.21±3.73c	326.12±2.20c
1-Butanol,3-methyl-, acetate	123-92-2	1143	440.07±1.73g	388.04±3.65f	201.63±3.19a	647.81±8.17h	437.71±3.56g	231.83±0.56b	304.84±0.49d	280.14±18.29c	365.67±2.97e	205.38±2.99a
2-Propenoic acid, butyl ester	141-32-2	1189	8.15±0.02d	7.85±0.01a	7.87±0.02a	7.86±0.02a	7.98±0.02c	8.26±0.02e	7.93±0.02b	7.87±0.02a	8.13±0.07d	7.84±0.01a
Hexanoic acid, ethyl ester	123-66-0	1240	423.32±15.52g	200.62±2.46c	243.66±6.71d	164.72±4.00b	307.70±4.55e	514.27±3.35h	361.79±6.77f	123.67±3.12a	208.04±4.00c	168.36±2.78b
Acetic acid, hexyl ester	142-92-7	1270	17.28±0.22c	28.34±0.48f	15.39±0.51b	17.47±0.27c	21.04±0.25d	16.11±0.66b	12.13±0.07a	32.24±1.27g	34.28±1.01h	24.89±0.06e
Benzoic acid, methyl ester	93-58-3	1631	11.29±0.02b	11.26±0.03a	11.26±0.02a	11.23±0.01a	11.23±0.00a	11.25±0.01a	11.24±0.00a	11.25±0.03a	11.34±0.02c	11.26±0.01a
Methyl salicylate	119-36-8	1747	16.50±0.15c	16.78±0.41c	14.61±0.46a	16.27±0.03c	16.63±0.14c	19.40±0.22d	20.41±0.32d	15.54±0.62b	19.92±0.21d	16.74±0.08c
1-Pentanol,4-methyl-	626-89-1	1314	100.27±8.18g	41.75±0.21d	45.79±0.40d	6.53±0.25a	51.38±0.28e	55.25±0.34ef	57.66±0.90f	30.59±1.15b	42.01±0.45d	35.72±0.53c
1-Octen-3-ol	3391-	1430	815.28±77.27h	458.48±32.51e	540.66±14.82f	411.19±4.55d	436.55±7.16de	776.62±2.67h	638.29±10.15g	56.89±0.33a	275.90±3.10c	223.09±3.90b

Volatile compounds	CAS	LRi	Wine samples									
			CK	JJ2D	JJ4D	JJ6D	LK2D	LK4D	LK6D	BX2D	BX4D	BX6D
	86-4											
1-Hexanol,2-ethyl-	104-76-7	1484	0.55±0.21b	1.97±0.11e	0.00±0.00a	0.00±0.00a	1.08±0.03d	0.00±0.00a	0.00±0.00a	0.83±0.22c	0.00±0.00a	0.00±0.00a
1-Octanol	111-87-5	1560	22.43±1.51g	6.46±0.15b	15.64±0.28e	11.01±0.14d	9.54±0.40c	18.31±0.14f	17.61±0.20f	3.53±0.32a	9.25±0.05c	6.15±0.03b
1-Nonanol	143-08-8	1673	12.46±0.37g	7.35±0.06c	7.28±0.32c	9.50±0.29e	8.81±0.15d	12.88±0.10h	9.87±0.11f	4.31±0.22a	6.21±0.03b	4.62±0.10a
Heptanal	111-71-7	1185	16034.09±818.78e	14123.11±184.34d	12441.24±35.08c	5437.91±94.60a	14062.96±54.14d	12221.04±148.52c	11397.04±316.79b	15531.33±179.45e	13655.07±12.59d	12120.01±184.63c
Octanal	124-13-0	1297	25.38±1.24b	28.08±0.13c	32.58±0.16e	15.41±0.17a	44.59±0.04h	39.75±0.05g	31.18±0.74d	27.13±0.23c	38.05±1.32f	27.21±0.34c
2-Heptenal, (Z)-	57266-86-1	957	427.29±4.44g	320.99±3.42d	362.22±2.22e	163.95±2.11a	530.62±7.82i	744.93±1.73j	481.29±3.30h	415.77±11.01f	265.10±4.02c	175.82±1.36b
Nonanal	124-19-6	1071	222.42±9.28h	109.84±0.51e	93.51±3.24d	56.97±1.05a	94.20±1.66d	88.34±3.05d	65.38±0.86b	146.60±8.66g	131.67±1.03f	75.07±0.20c
2-Octenal, (E)-	2548-87-0	1430	55.10±12.11d	24.42±0.51ab	33.25±0.97b	192.62±3.52e	26.23±0.59ab	29.26±0.35b	46.95±1.91c	29.41±0.45b	27.91±0.42ab	21.39±0.26a
2,4-Heptadienal,	4313-	1461	31.86±1.22e	16.61±0.02d	13.54±0.47b	9.72±0.27a	12.84±0.02b	10.82±0.18a	10.29±0.06a	16.66±0.34d	15.41±0.10c	13.57±0.46b

Volatile compounds	CAS	LRi	Wine samples									
			CK	JJ2D	JJ4D	JJ6D	LK2D	LK4D	LK6D	BX2D	BX4D	BX6D
(E, E)-	03-5											
Benzaldehyde	100-52-7	1530	52.56±1.13h	36.48±0.25d	41.90±1.07f	63.57±0.34i	27.99±0.22b	25.59±0.33a	31.11±0.01c	30.77±0.17c	50.56±0.91g	39.04±0.10e
2,4-Nonadienal, (E, E)-	5910-87-2	1712	17.31±0.40c	16.80±0.17ab	16.49±0.07a	16.49±0.08a	16.77±0.07ab	17.02±0.11b	16.93±0.03ab	16.73±0.14a	16.88±0.10ab	16.56±0.14a
β-Myrcene	123-35-3	1155	27.83±0.35a	29.17±0.65b	28.73±0.27b	27.71±0.23a	28.68±0.13b	28.47±0.31ab	28.35±0.10ab	27.86±0.84a	28.95±0.71b	27.92±0.10ab
α-Phellandrene	99-83-2	1149	22.97±0.03a	22.98±0.01a	22.95±0.01a	22.98±0.02a	22.98±0.01a	22.98±0.01a	22.98±0.02a	22.97±0.03a	22.97±0.01a	22.96±0.00a
D-Limonene	5989-27-5	1030	23.30±0.05ab	23.32±0.11ab	23.17±0.04a	23.12±0.08a	23.38±0.01b	23.27±0.02ab	23.52±0.07b	23.37±0.19b	23.45±0.20b	23.20±0.07ab
p-Cymene	52462-29-0	1020	36.23±0.11a	36.34±0.01a	36.21±0.00a	36.26±0.02a	36.28±0.01a	36.34±0.02a	36.28±0.06a	36.25±0.07a	36.25±0.01a	36.19±0.01a
1-Octen-3-one	4312-99-6	1317	15.74±0.94d	10.78±0.10b	11.00±0.38b	9.64±0.02a	10.12±0.02a	12.36±0.11c	9.96±0.00a	12.10±0.17c	11.97±0.12c	11.80±0.13c
Caryophyllene	87-44-5	1622	28.44±0.03b	28.90±0.15b	29.50±0.42c	34.05±0.53d	29.87±0.10c	37.84±0.49e	37.86±0.01e	26.55±0.09a	26.80±0.02a	26.73±0.08a
Carvone	6485-40-1	1245	11.84±0.05g	5.69±0.12c	8.18±0.07d	5.67±0.03c	5.30±0.08b	9.24±0.13e	9.80±0.00f	5.04±0.05a	8.35±0.34d	5.71±0.12c

Volatile compounds	CAS	LRi	Wine samples									
			CK	JJ2D	JJ4D	JJ6D	LK2D	LK4D	LK6D	BX2D	BX4D	BX6D
2-Buten-1-one,1-(2,6,6-trimethyl-1,3-cyclohexadien-1-yl)-	23696-85-7	1362	37.74±1.31cd	44.07±0.71f	36.59±0.94c	22.65±1.10a	45.47±0.37g	54.79±0.11h	41.66±0.91e	39.11±0.12d	45.56±0.99g	34.82±0.61b
Pyrazine, methyl-	109-08-0	1264	15.26±0.17a	19.45±0.39f	15.69±0.21b	14.93±0.10a	15.94±0.06bc	18.36±0.05e	20.22±0.09g	16.17±0.20c	16.77±0.24d	15.78±0.20bc
2H-Pyran, tetrahydro-4-methyl-2-(2-methyl-1-propenyl)-	16409-43-1	1337	5.70±0.01b	5.65±0.00a	5.68±0.01b	5.64±0.00a	5.69±0.00b	5.69±0.01b	5.67±0.01b	5.67±0.02b	5.75±0.02c	5.67±0.01b
Linalool	78-70-6	1540	0.99±0.07c	2.84±0.00e	0.48±0.01b	0.00±0.00a	2.32±0.20d	2.85±0.04e	0.00±0.00a	3.72±0.34f	5.57±0.15g	2.32±0.15d
Terpinen-4-ol	20126-76-5	1182	12.66±0.10c	12.63±0.09c	11.64±0.04a	11.56±0.02a	11.80±0.04ab	12.06±0.03b	11.72±0.09ab	11.94±0.19b	12.00±0.11b	11.97±0.11b
α-Terpineol	98-55-5	1688	12.55±0.28a	13.34±0.10c	12.48±0.23a	12.19±0.15a	13.01±0.02b	13.80±0.05cd	14.24±0.13d	13.53±0.36c	14.04±0.04d	12.87±0.05b
Citronellol	106-22-9	1750	20.43±1.05e	0.00±0.00a	7.19±0.41b	0.00±0.00a	0.00±0.00a	12.29±0.13c	13.31±0.24d	0.00±0.00a	7.75±0.14b	0.00±0.00a
Geraniol	106-24-	1860	62.14±2.86a	145.43±2.01g	88.38±1.01c	70.25±1.45b	151.91±0.58h	126.48±1.87f	117.72±0.56e	121.09±1.38e	101.38±4.13d	90.10±1.34c

Volatile compounds	CAS	LRi	Wine samples									
			CK	JJ2D	JJ4D	JJ6D	LK2D	LK4D	LK6D	BX2D	BX4D	BX6D
	1											
p-Mentha-1,8-dien-7-ol	536-59-4	1287	21.95±1.02b	37.01±0.24g	22.74±0.47bc	14.50±0.06a	23.57±0.16c	23.79±0.40c	25.14±0.41d	45.56±0.76h	31.42±0.88f	28.74±0.56e
Ethyl Acetate	141-78-6	896	164.16±8.36a	341.99±6.27bc	620.59±10.94d	2294.18±179.88e	335.76±2.83bc	540.92±1.29d	574.85±2.81d	285.57±2.19b	385.75±3.96c	298.04±7.66bc
1-Butanol	71-36-3	1116	12.27±0.48g	10.06±0.09cd	10.76±0.01e	7.85±0.14a	11.30±0.00f	10.36±0.03d	9.42±0.03b	12.02±0.20g	10.80±0.19e	9.78±0.01c
1-Butanol, 3-methyl-	123-51-3	1217	899.69±38.19f	804.28±5.66e	708.12±2.39d	382.78±2.32a	804.63±0.46e	768.23±0.21e	679.27±4.04c	892.83±2.29f	785.25±1.65e	641.31±3.63b
3-Hexen-1-ol, (Z)-	928-96-1	1383	9.54±0.05g	9.12±0.11f	7.67±0.17d	4.10±0.02a	10.92±0.03h	11.59±0.20i	7.91±0.02e	8.00±0.07e	7.49±0.00c	5.34±0.12b
Phenylethyl Alcohol	60-12-8	1920	454.97±16.34e	459.65±13.17e	297.52±1.14bc	78.13±1.62a	387.74±0.69d	313.13±5.00c	358.88±2.02d	454.85±23.81e	370.21±12.17d	281.52±7.34b

Note: Different letters represent significant differences between the wine samples according to Duncan's test ($p < 0.05$). LRi values representing the Linear Retention Index were determined on a DB-Wax column.
